# Semen Parameters of Fertile Guinea Pigs *(Cavia porcellus)* Collected by Transrectal Electroejaculation

**DOI:** 10.3390/ani10050767

**Published:** 2020-04-28

**Authors:** Fernando Benavides, Peter Sutovsky, Verónica López, Chelsey Kennedy, Luisa Echevarría

**Affiliations:** 1 Laboratory of Animal Reproduction, Zootechnic and Veterinary Medicine School, Cayetano Heredia Peruvian University, Honorio Delgado 430, San Martín de Porres, Lima 15102, Peru; 2 Division of Animal Science, University of Missouri, Columbia, MO 65211, USA; 3 Departments of Obstetrics, Gynecology and Women’s Health, University of Missouri, Columbia, MO 65211, USA

**Keywords:** electroejaculation, flow cytometry, guinea pig, parameters, semen, ubiquitin

## Abstract

**Simple Summary:**

The guinea pig is an important livestock species in some South American countries. Peruvian guinea pig exports are still increasing since 2000. Peru has made genetic improvements on the species since 1986. However, there are few advances in reproductive biotechnology. Thus, there is a lack of information about harmless, efficient semen collection techniques in the species. Consequently, the selection of fertile males takes a long time due to the absence of validated seminal parameter standards. For that reason, it is necessary to set semen reference values through an objective electroejaculation technique for guinea pigs. This study describes semen parameters of fertile males and validates a novel semen collection technique for the species. These values will serve as a reference to detect infertile males and to select the best males for breeding purposes, improving the reproductive performance on farms.

**Abstract:**

The guinea pig, as a livestock species, is still developing and growing throughout Peru and neighboring countries, as reflected by its increasing export since 2000. However, the selection of proven fertile males is tedious due to the absence of seminal parameter standards and the lack of safe semen collection techniques. Thus, pregnancy detection or live births are required for males’ selection. The purpose of this study was to describe the qualitative and quantitative semen parameters of fertile guinea pig males, to set reference values, and to validate a novel electroejaculation technique for the species. Semen was collected at weekly intervals from sixteen fertile males. Four transrectal electroejaculations were performed per male with 95% successful collections, yielding 39 viable semen samples. Seminal characteristics were as follows: pH 7.0 ± 0.13; ejaculate volume 0.67 ± 0.55 mL; sperm motility 90.81 ± 6.64%; sperm concentration 36.7 ± 28.41 × 106 sperm/mL; sperm count 20.09 ± 17.56 × 106 sperm/ejaculate; percentage of abnormal morphology 18.26 ± 8.52%; and percentage ubiquitinated spermatozoa 5.57 ± 6.28%. These values will serve as a reference to detect best breeding and infertile males rapidly. The described techniques are reproducible by commercial producers.

## 1. Introduction

The guinea pig *(Cavia porcellus)* is an important meat-producing livestock species in Peru and in other central/south American countries, capable of efficient feed conversion into meat [1,2]. In Peru, accumulative exports between 2001 to 2007 were worth $306,864 US dollars; more recently, 2016 exports were more than 15,000 tons, a considerable amount for a nontraditional product, motivating producers to be more competitive [3,4]. Andean women are traditionally in charge of guinea pig production, giving them work and a source of income [5]. The guinea pig head count in Peru is the highest in South America, with 12,695,030 animals [4]. Other producing countries with important guinea pig counts are Ecuador with 5,067,049 animals [6], Colombia with 1,292,244 animals [7], and Bolivia with 650,000 animals [8]. 

The guinea pig industry depends upon the selection of animals with superior production traits and reproductive performance. Thus, genetic improvement is limited by the use of reproductively untested males due to the difficulty of semen collection and the natural irritable disposition of this animal. Moreover, once males finish their productive life, the valuable genetic material is lost [2]. Thus, the industry seeks to identify the best fertile males faster through semen analysis instead of waiting for pregnancy and birth rates and to develop reproductive biotechnologies such as artificial insemination (AI).

Electroejaculation is a physical method of ejaculatory induction allowing for repeated semen evaluation of individual animals and cryobanking of semen for AI as well as for utilizing males unable to breed naturally [9]. There are few and mostly old reports about guinea pig electroejaculation [2,10,11]: 

In 1959, Scott and Dziuk [11] employed the electroejaculation method using increasing electrical pulses from zero to 5 or 10 peaks of alternating current volts. They used a bipolar rectal electrode inserted up to the fourth lumbar vertebra. However, their report does not detail the method’s success rate. Freund [10] used 12-volt square waves every 3 s followed by 12 s of rest. He employed two separated electrodes; one was inserted in the rectum, and the other was a disc positioned at the second and third lumbar vertebrae. However, the report did not specify the success rate. More recently, Garcia and Moncayo [2] employed a similar technic as Freund, with two separated electrodes positioned at the same point, although the rectal stimuli were 20 or 25 volts and a 30-volt electric discharge every 15 s. Like the aforementioned reports, the authors did not mention the success rate of the method.

In Peru, an old electroejaculation method was developed using indefinite electrical pulses of 8 alternating current volts every 3 s followed by 10 s of rest, using a 7-cm bipolar rectal electrode, with 90% collection success [12]; however, we were not able to replicate this technique with acceptable results. Thus, the aforementioned work does not specify the organ or nerve to be stimulated, only approximate subjective referential topographic areas.

Early sensory evaluation of guinea pig semen characteristics by Freund [10] revealed average sperm volume of 0.5 mL, ranging from 0.2 mL to 1 mL. Later, García and Moncayo [2] found volume variations from 0.6 to 0.8 mL. Similar to boars and stallions, guinea pigs were reported to ejaculate in three seminal fractions: the pre spermatic fraction, the spermatic (spermatozoa rich) fraction, and the gel fraction. About microscopic sperm evaluations, García and Moncayo [2] measured average sperm concentration at 25 × 10^6^ spermatozoa/mL while Scott and Dziuk [11] and Freund [10] reported values of 5 to 235 × 10^6^ spermatozoa/ejaculate and 2.9 to 35 × 10^6^ spermatozoa/ejaculate, respectively. Freund [10] also reported 5% average of spermatozoa with morphologic abnormalities. Regarding progressive motility, García and Moncayo [2] found 90% average while Freund [10] reported 66%. Such disparate findings demonstrate a wide variation of seminal characteristics of guinea pigs, being more prominent between individual males than between multiple collections from the same male, according to Freund.

Ubiquitin (UBB) is a chaperone protein present in the mammalian testis and epididymis that binds to other proteins destined for recycling by proteolysis. It attaches covalently to substrate’s lysine residues to mark defective or outlived proteins for proteolytic degradation [13]. According to Sutovsky [14], the percentage of ubiquitinated spermatozoa in semen reflects semen quality and fertility in several animal species as well as humans. Likewise, this method has been used to validate a dual ubiquitin-PNA(Peanut agglutinin lectin)-based bull sperm assay by Odhiambo [15], using the EasyCyte Plus flow cytometer. However, there are no data on the ubiquitination of guinea pig spermatozoa by microscopical or flow cytometric analysis. While the ubiquitin-based sperm flow-cytometric assay has been used in a number of human and ungulate studies [16], the only instance the ubiquitin flow cytometry was applied to a rodent was a reprotoxic study in rats. In this report, the average percent of defective, ubiquitinated spermatozoa was less than 10% in the control, presumably fertile males compared to as much as 40% in males affected by experimental reprotoxic intervention [17]. Such levels are comparable to sperm ubiquitin values reported in the present guinea pig study (less than 10% on average but as high as 20% in some males/ejaculates). 

The purpose of this study was to describe and set reference values of the qualitative and quantitative semen parameters of fertile guinea pig males while also validating a novel electroejaculation technique suitable for AI. Based on this knowledge, producers will be better able to select fertile males and to reject infertile males with poor semen quality. Such selection can accelerate genetic progress and can maximize the reproductive potential of female guinea pigs.

## 2. Materials and Methods

### 2.1. Animal Selection

A total of 16 fertile Peruvian male guinea pigs *(Cavia porcellus*), with a minimum of 7 healthy litters, were maintained at the Guinea pig Experimental Station La Molina of the National Institute for Agricultural Innovation (INIA) located in La Molina district, Lima, Peru (Coordinates 12°04’35.8” S 76°56’42.9” W). The station belongs to the National Guinea pig Program of the Peruvian Ministry of Agriculture and Irrigation. The animal management at this station is within the established standards of the Code of Practice for the Housing and Care of Animals Used in Scientific Procedures [18]. Guinea pig were maintained under identical feeding conditions: corn *(Zea mays)* (fresh forage including stalk and cob), commercially balanced food for guinea pig (18% of total protein (TP) and 2.8 megacalories of metabolizable energy (ME)), and water. The average environmental temperature during the study was 21.3 ± 1.74 °C, with 70% relative humidity and natural lighting. As an inclusion criterion, we used adult animals with healthy offspring, weight over 1.1 kg, without evidence of systemic or andrological disorders. Andrological examination, electroejaculation, sampling, and semen evaluation were performed at the Laboratory of Animal Reproduction, Zootechnic, and Veterinary Medicine School, Universidad Peruana Cayetano Heredia, Lima, Peru.

### 2.2. General and Andrological Examination

Recorded data included identification tag number, age, weight, rectal temperature, body condition, hair appearance, possible nasal or ocular secretions, and any other injuries. Andrological evaluation of each animal considered testis consistency/texture/palpation, scrotum integrity, and the presence of gross anatomical defects. Likewise, the penis was examined for the absence of prepuce adhesions, abscesses, or other injuries in addition to the absence of foot or limb lesions. Affected animals and those with records of infertility were excluded.

### 2.3. Animal Handling

The study was approved and monitored by designated Doctor of Veterinary Medicine (DVM) representative of the Zootechnic and Veterinary Medicine Faculty of Cayetano Heredia Peruvian University, strictly adhering international ethics standards set by the Laboratory Animal Science Association (LASA) [19].

The success of the electroejaculation procedure depends on minimizing stress. For that reason, guinea pig care was crucial and based on the guidelines for transport of laboratory animal established by LASA [19], as follows: 

Only animals presenting normal behavior, without apparent health problems, and with normal physiologic constants were transported. All guinea pigs were transported in the same boxes used at the Experimental Station farm for handling those animals at all times; they were transported gently and at slow speeds, avoiding sudden movements or extensive vibrations. Only one male per box was transported. The box was 80 × 60 × 40 cm W × L × H; it had multiple openings maximizing visibility and ventilation and a sponge bed. 

We picked up the guinea pigs at 7 a.m. before first feeding to avoid sedation accidents in the lab. Guinea pigs were transported by car; in the back seat; with 20 °C air conditioner; without music; and covered by a thin, white, porous blanket; they traveled next to the veterinary assistant who checked them every five min. The trip was completed at a moderate speed and by avoiding any sudden movements or potholes. 

Upon arriving in the laboratory, guinea pigs were taken to the soundproofed procedure room. Semen was only collected from guinea pigs under deep sedation, constantly evaluating vital signs such as body flaccidity, visual or manipulation reflex, retraction of the hind limbs after stretching, slight palpebral reflex, and mainly normal physiological constants for sedation. 

After semen collection, guinea pigs were placed in their transport box with a fresh sponge bed, checking their vital signs every 5 min. After waking up, animals were transported back the same way to their home farm. This procedure was followed exactly at every sampling. After four weekly collections, guinea pigs were placed in quarantine to monitor their health and behavior.

### 2.4. Semen Collection by Electroejaculation

#### 2.4.1. The Electroejaculator Device

The device emits 6 volts of sine alternating current which is emitted in the form of waves from −6 to +6 volts to 50 cycles per s. It has a timer that automatically controls current emission and break cycles (3 emission seconds and 10 of rest) and a red-light indicator of current emission. The current is emitted through a rigid bipolar longitudinal probe made of steel, 12 cm long × 0.6 cm in diameter. Both poles are located on the same probe. The first is at the tip and is 0.6 cm long, while the second pole is 11.6 cm long. Both poles are separated by a 3-mm strip of insulating material.

#### 2.4.2. Sedation Protocol and Electroejaculation

Before sedation, feces were removed from the rectum and perineal zone and the penis was wiped dry. The feces were removed by gently massaging and stimulating the anus with saline gel. Sedative treatment consisted of a single dose of ketamine hydrochloride (40 mg/kg Intramuscular [20]). Thirty min later, investigators made sure that the animal was completely sedated by not responding to physical or sound stimulation. Immediately before electroejaculation, the animal was positioned on a flat table and the skin was marked over the point where the rectal probe tip would be located. This was the intersection point of an imaginary line that connects both external iliac tuberosities with the middle line demarcated by the rectum and the distal part of descending colon. In that intersection, the hypogastric nerves, responsible for seminal emission, can be stimulated. To identify this nerve, ten animals were previously dissected to observe its position and path. To identify how deep to introduce the first pole of the rectal probe (the tip), the external topographic spot was located and matched internally to the point where the two hypogastric nerves were close enough to the rectum. 

The rectal probe from the electroejaculator was introduced in the rectum previously lubricated with conductor saline gel, and the penis was introduced into the collector tube. Stimuli of six sine wave alternate current volts and 50 hertz frequency were conducted through the bipolar rectal probe with 3 second of stimuli alternating with 10 seconds of rest. Continuous stimulus cycles were applied until the complete gel-free semen fraction was obtained. A maximum of 15 cycles per animal were needed. In each stimulus cycle, the animal showed a reflex, stretching the hind limbs. During ejaculation, there was a slight reflection of pelvic movement. Both reflexes ceased after ejaculation.

#### 2.4.3. Semen Collection

Semen samples were collected in a 3-mL graduated glass tube containing only 0.5 mL of 3% sodium citrate diluted in twice-distilled water (dilutor) previously warmed in a water bath to 39 °C to avoid semen coagulation [21,22,23,24]. During ejaculation, an assistant held the animal, mixed semen with diluent, and removed the gel fraction with a fine spatula to collect the entire pre-spermatic and spermatic fractions.

### 2.5. Semen Evaluation

#### 2.5.1. Macroscopic Evaluation

Volume was measured in the same small tube where the sample was collected. Volume was calculated by subtracting the initial volume (diluent only) from the final volume (dilutor volume plus the semen volume). pH was measured using pH paper indicator once the semen was collected [22] but before it was mixed with the dilutor. Semen color and neutral pH levels indicated that samples were not contaminated with blood (color), urine [25] (pH), and/or other detectable contaminants. 

#### 2.5.2. Microscopic Evaluation

Motility was evaluated immediately after the collection. After mixing semen with the dilutor, a drop of semen was placed on a slide prewarmed to 39 °C on a heated stage of the microscope and evaluated immediately, twice at × 400 magnification. Results were expressed as the percentages of progressively motile or immotile spermatozoa [26]. To be considered motile, spermatozoa were expected to swim in progressive and curvilinear manner typical of the species [10].

#### 2.5.3. Sperm Count and Concentration

A total of 10 µL of sample was added to 90 µL of formal saline solution [27]; 10 µL of the new dilution was placed in the Neubauer Chamber to standardize the sample and was counted after 10 to 15 min, according to Kvist et al. [26]. Sperm concentration took into account volumes of formal saline dilution factor and sodium citrate dilution factor. The entire process was repeated for each sample to confirm results. Sperm concentration was expressed in millions (106) per mL. Total sperm count was calculated from sperm concentration and semen volume.

#### 2.5.4. Morphology

An Eosin-Fast green stain made with 1 part of 1% aqueous eosin B, 2 parts of 1% aqueous fast green FCF, and 2.7 parts of 95% ethanol was used to evaluate sperm morphology. A total of 20 µL of semen mixed with the dilutor was spread and dried on a slide and overlaid for 10 s with 98% methanol fixative and then for 2 min with eosin-Fast green solution. A total of 200 spermatozoa was evaluated twice to increase accuracy under a light microscope at × 400 magnification [26]. The results were expressed as percentage of abnormal spermatozoa based on the classification of sperm abnormalities found in other mammals, with a special reference to rabbits [28].

#### 2.5.5. Sperm Ubiquitin Assay

A 100 µL volume of diluted semen with sodium citrate was placed in a cryovial with 400 µL of 2% formaldehyde and allowed to sit for 40 min. The sample was homogenized and centrifuged at 1800× *g* for 6 min, after which the supernatant was removed and replaced with Phosphate-buffered saline (PBS). Washing was repeated twice. 

Samples were sent by air transport and analyzed at the Division of Animal Sciences, University of Missouri, Columbia, MO, USA. The pellet was divided into two equal parts, one for the labeling with anti-ubiquitin antibody and the other for negative control. To block nonspecific reactions, the pellet was incubated for 30 min in 1 mL of 5% normal goat serum (NGS; Sigma-Aldrich Corp. St. Louis, MO, USA) in PBS. After centrifugation and supernatant removal, a 200-µL volume of anti-ubiquitin antibody KM-691 (Kamyia Biomedical Comp., Seattle, WA, USA; dil. 1/100 in PBS/1% NGS) was added to the pellet, mixed, and incubated for 40 min at room temperature. Next, the sample was washed by resuspension in 10 mL of PBS/1% NGS and centrifuged and then incubated with 200 µL of goat anti mouse IgM-FITC (Fluorescein isothiocyanate) (Zymed Labs, San Francisco, CA, USA; dil. 1/200). Finally, the spermatozoa were washed with 10 mL of pure PBS and resuspended in 500 µL pure PBS for immediate flow cytometric analysis.

**Figure 1:**
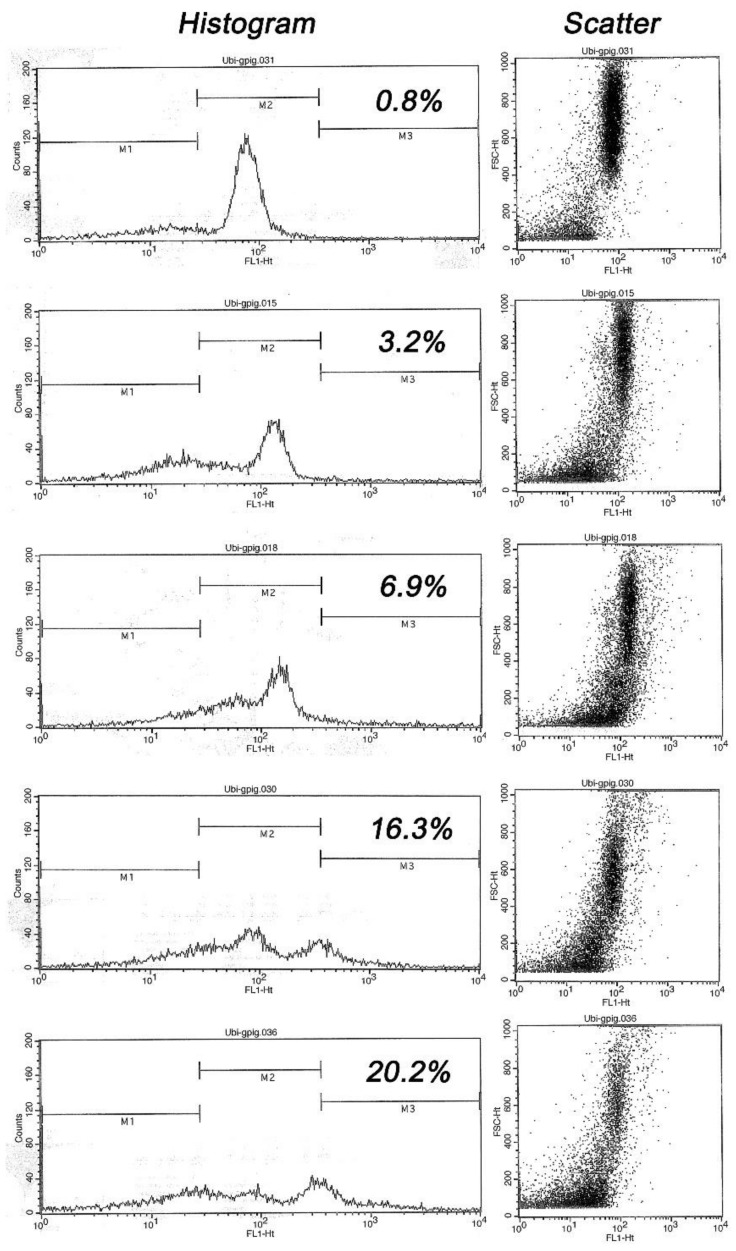
Flow cytometric histograms of ubiquitin-induced fluorescence in five males, ranked top-to bottom from lowest to highest sperm ubiquitin content (% ubiquitinated spermatozoa within marker area M3 is shown): Note a progressive shift of histogram towards M3 area, resulting in a distinct second peak in high ubiquitin samples. Such a trend is mirrored by scatter diagrams of visible light in the right column (each dot represents one cell/flow cytometric event), where the events in the lower left corner represent undesirable abnormal cells and debris while the focus of dots spanning from scatter center to upper right corner represents the cells of normal/desirable size and shape.

Flow cytometry data and analysis was performed in the EasyCyte Plus sperm flow cytometer (IMV Technlogies, L’Aigle, France) essentially as described by Odhiambo et al. [15]. Ubiquitin levels were measured in 5000 cells/sample and recorded as scatter plots and histograms. After the samples were measured, the histograms were standardized and the markers were ordered for each specific population. Next, the sample was divided into three subpopulations using a standardized process of conversion and differentiation based on the scatter plots (Figure 1). 

Flow cytometry result readouts were subdivided according to fluorescence levels and the percentages of spermatozoa inside each individual marker area of the histogram. Marker 1 (M1) contained the cells with relatively low fluorescence, including cellular detritus and spermatozoa with low or no ubiquitin; Marker 2 (M2) represented spermatozoa with an ubiquitin range close to the mean of ubiquitin induced fluorescence typical of morphologically normal spermatozoa; and Marker 3 (M3) represented spermatozoa with higher fluorescence levels corresponding to morphologically abnormal spermatozoa. 

These subpopulations were analyzed by using the CytoSoft software of the EasyCyte Plus instrument. Results in this study were expressed as percentage of highly ubiquitinated spermatozoa (sperm population within marker M3).

### 2.6. Data Analysis

For the purpose of this study, the first ejaculate from each animal was excluded because first collections typically had low volume and poor sperm quality not representative of all ejaculates collected from individual animals. Thus, the first criterion for inclusion in data analysis was that the animal had at least two successful collections, defined as semen samples with motile spermatozoa and sperm count of more than 1 × 10^6^ spermatozoa/ml. Likewise, subsequent collections with sperm concentration lower than 5 × 10^6^ spermatozoa/mL were also eliminated from the analysis because of their poor sperm quality, and this was considered the second criterion for inclusion.

We further applied a third criterion, i.e., the application of “Box and Whisker Plots” of concentration values, excluding guinea pigs with values over 200 × 10^6^ spermatozoa/mL. Altogether, this strategy allowed us to establish more uniform spermatic values ranges according to the objective of the study. After discarding all animals within the exclusion criteria, we performed all analyses in 16 animals.

Data was analyzed by descriptive statistics including media, minimum and maximum values, and standard deviation; variance analysis was used to capture variables of semen evaluation in relation with the number of ejaculations and Duncan test for mean differences with 0.05 signification level. Likewise, we used a correlation test between obtained values and the number of successive ejaculates with 0.05 signification level. Statistical results were obtained using the SPSS program.

## 3. Results

### 3.1. Semen Collections

A total of 64 collection attempts from 16 animals was made (four attempts/animal), of which 61 successful sperm-containing ejaculates and 3 aspermic ejaculates were obtained (95% efficiency).

From the 61 successful ejaculates, thirty-nine ejaculates from 16 animals were evaluated by the exclusion criteria described above. We used 3 ejaculates from each of 10 animals, 2 ejaculates from each of 3 animals, and one ejaculate from each of the remaining 3 animals. All of them were clot free.

### 3.2. Seminal Characteristics

PH values ranged from 7 to 8, with a mean pH of 7 ± 0.13 (Table 1). The mean found for ejaculate volume was 0.67 mL ± 0.5. There was a tendency to increase volume in successive weekly ejaculations from each animal, although there was no significant difference, mean, or correlation between average seminal volume and successive weekly ejaculations (Table 2).

Average motility was 91% ± 6.6, while mean concentration was 36.7 × 10^6^ ± 28.4 spermatozoa/mL.

The percentage of total abnormalities was 18.26% ± 8.5, and the percentage of ubiquitinated spermatozoa was 5.57% ± 6.3. Other parameters are detailed in Table 1. 

Mean of sperm motility, sperm concentration, and the percentage of ubiquitinated spermatozoa remained constant within a male in successive weekly ejaculations. Similarly, the average total numbers of ejaculated spermatozoa and morphological abnormalities did not show statistically significant differences between successive weekly ejaculations but showed a tendency to increase (Table 2).

Due to the prevalence of acrosomal abnormalities and to facilitate analysis, we classified sperm defects as follows: abnormal acrosome (deformed or destroyed); absent acrosome; and other abnormalities which included macrocephalic and microcephalic heads, broken tails, midpiece abnormalities (thickening, eccentric sperm tail attachment, cytoplasmic droplet, and corkscrew defect), and sperm tail principal piece abnormalities (knotting, coiled, whip tail, and bent tail). We found that only the percentages of absent acrosome and misshapen acrosome showed a tendency to increase with consecutive collections but was not statistically significant. Frequencies of other abnormalities remained constant (Table 3).

## 4. Discussion

### 4.1. Electroejaculation Efficiency

The main difference of this electroejaculation method is the identification of the nerve to stimulate (Hipogastric nerve), for which the function is to induce the seminal emission that triggers the ejaculation. As Birnabaum and Hall [29] reported in rats, the authors observed that, if the first pole (the tip) of the rectal probe is not in the correct point, the success of the collection is affected, as are the seminal values. In previous reports of electroejaculation in guinea pigs, there was no mention of the organ or nerve stimulated during the electroejaculation; for this reason, we believe these values are not reliable; thus, it is possible that ejaculates were obtained by stimulating the responsive regions by trial and error. This is the reason why our collection rate is high, even without high voltage and when using sedation (unlike previous reports), and why our seminal values are reliable, being similar or better than previously reported.

We have not detected a major influence of our electroejaculation technique on semen parameters. Dooley and Pineda [30] demonstrated that higher voltages during feline electroejaculation produced abrupt increases in semen osmolarity. Likewise, changes in electroejaculated bulls’ semen osmolarity coincided with disrupted plasma membrane integrity, acrosomal damage, and acrosomal loss [31]. In humans, semen hyperosmolarity has a stimulatory effect on acrosomal exocytosis [32]. Thus, it is plausible that the increase in acrosomal damage and acrosomal loss in our study could be due to osmolarity changes produced by the voltage used. Future studies will analyze the effect of different voltages on osmolarity values and acrosomal integrity in successive guinea pig electroejaculation. 

### 4.2. Macroscopic Parameter Values and Variability:

As Garcia and Moncayo reported [2], we found three seminal fractions: pre-spermatic, spermatic, and gel fraction; however, the gel fraction was more viscous than found in stallion and boar. Furthermore, in our study, the fractions in few cases were intermixed, which made it difficult to separate, possibly due to the abrupt stimuli induced by the electric discharge, as described in rats [29] and mice [9]. 

According to Birnabaum and Hall [29], to achieve proper separation of spermatic and gel fractions in rats requires correct voltage and frequency and the proper probe position inside the rectum. That could be the reason why we were able to separate ejaculate without intermixing them. On the contrary, other reports in rodents described two intermixed fractions, gel and non-gel fraction, possibly due to the voltage or frequency applied [33,34].

The non-gelatinous fractions tend to coagulate after being expelled; so, for proper evaluation, it is necessary to collect it in a sodium citrate solution that prevents clot formation [11,12]. As reported in stallion [27], rats [29], and mice [9], gel fraction has a negative effect on spermatozoa, immobilizing them and coagulating the semen. For this reason, it is necessary to separate it from the spermatic fraction.

Seminal volume average (0.67 mL ± 0.5) shows wide variation and a tendency to increase in successive ejaculations (Table 2), as Freund reported [10]. This wide volume variation is likely due to a better hypogastric nerve response to successive electroejaculations and possibly a reduction of gel fraction because this normally induces a rapid gelling of the spermatic fraction. In general, our macroscopic findings agree with those reported by Garcia and Moncayo [2], whose studies provided guidance for artificial insemination programs. 

Comparing with other rodent reports, average guinea pig semen volume, considering only gel free fractions, is lower than in rabbits [35] but higher than in chinchillas [36], rats [34,37], and mice [33]. A wide range of variability between individual animals was reported in those species, just as we found in guinea pigs. It could be due to interspecific differences in body weight and the size of accessory glands.

On the other hand, pH values in our study were uniform within and between individual animals, reflecting that pH measurements made immediately after collection, before lactic acid increase, and consistent with the absence of urine contamination [25] or infections [27]. We thus have confidence for future use of our electroejaculation technique in commercial AI programs. 

### 4.3. Microscopic Parameters and their Variability

Motility values reported here (90.86% ± 6.64%) are similar to those of García and Moncayo [2] who, similarly, selected healthy, fertile animals for insemination; however, Freund [10] found an average 66% sperm motility, likely due to different electroejaculation technique employed. Furthermore, if we consider other hystricomorphous rodents like aguti, average motility was 50.44% ± 4.44%, probably reflecting natural reproductive selection [38]. However, it should be considered the subjective nature of routine spermiogram; besides, motility measurement in guinea pigs is complicated by naturally occurring stacking of 5 to 7 spermatozoa and their normal curvilinear motility, as previously described [39].

Our measurements of sperm concentration (36.7 ± 28.4 million/mL) differ significantly from the average of 25 million/mL reported by García and Moncayo [2], possibly because they used a different electroejaculation technique in their study. On the other hand, the total number of ejaculated spermatozoa in the present study showed a similar variation as reported by Freund [10], i.e., 2.9 to 35.0 × 10^6^ spermatozoa per ejaculate, and by Scott and Dziuk [11], who reported a massive variation ranging from 5 to 235 million/ejaculate. This wide variation in the total number of ejaculated spermatozoa was due to variation in ejaculate volume and sperm concentration and, as aforementioned, by lack of selection of males based on semen quality. 

Percentages of spermatozoa with abnormal morphology in our study were greater than reported by Freund [10], although these were still within accepted standards for most species [27,40,41] including rabbits, rats, mice, and wild rodents [37,38,42].

The low average percent of ubiquitinated spermatozoa found in this study indicates that most sperm abnormalities observed in morphological analysis were not induced in the testis or epididymis. According to Sutovsky [14], there are no established reference values for ubiquitin-positive spermatozoa in guinea pigs; thus, it is not possible to compare sperm ubiquitin values with other rodent species due to different normal acceptable values studied previously. Likewise, the type of anti-ubiquitin antibody used to detect abnormal spermatozoa in different species varies and makes it difficult to extrapolate. According to Sutovsky [14], the best way to identify normal reference values for the species is by using healthy animals and a correct sampling procedure. For example, he proposes that, for bull spermatozoa, the acceptable percentage of ubiquitinated spermatozoa is below 30%.

### 4.4. Semen Quality at Successive Electroejaculations

This study confirmed the relationship between semen parameters and number of collections. The observed trend towards increased average semen volume in successive collections agrees with Freund [10]. This may be due to the once-a-week collection schedule that reduces coagulation and increased volume or proportion of non-gel fraction to gel fraction, as also reported by Vivanco et al. [12]. A possible reason is a desensitization of seminal vesicles to electric stimuli, which would reduce the gel fraction of semen.

Likewise, Birnabaum and Hall [29] collected multiple ejaculates and avoided the gel fraction by gradually decreasing voltage, resulting in an increase of zinc ions in semen, probably due to ejaculate volume and spermatozoa proportion. Therefore, a higher semen volume represents more seminal plasma enriched naturally with zinc, which has recently been shown to play a role in the regulation of mammalian sperm function and fertility [43,44]. However, in our study, the trend towards the increase of sperm volume was accompanied with unchanged sperm concentration and increase in the average total number of spermatozoa per ejaculate at successive collections in individual animals. 

The most likely reason for the progressive increase of spermatozoa with morphological abnormalities in successive ejaculations, particularly including the absence of acrosome and misshapen acrosome, was the post-ejaculation processing technique and the trend of increasing spermatozoa number. Acrosomes apparently suffer damage during the time period between ejaculation and semen evaluation, possibly because of slow gelling, as also observed in stallion and boar semen. Otherwise, if such abnormalities arose in the testis or epididymis, we would expect to see an increase of highly ubiquitinated spermatozoa, caused by the ubiquitin-dependent sperm quality control mechanism operating within the epididymal lumen [13]. 

During epididymal passage, abnormal spermatozoa, including those with defective acrosomes, are recognized and tagged with chaperone protein ubiquitin, which tags damaged proteins for recycling by 26S proteasome, lysosome, or autophagosome [45]. Even so, the average percentage of sperm ubiquitination in up to three consecutive ejaculates per male did not show significant differences, and sperm morphology remained within acceptable standards defined for other species [27,40,41], including rodents [37,38,42]. 

Finally, Table 1 and Table 2 demonstrate a greater variation of semen parameters between animals than among multiple collections from the same animal, as Freund [10] reported, using fertile males selected based on mating conception rates. Such consistent findings decades apart reinforce the view that males need to be selected for AI service by semen quality, which appears to be relatively constant in fertile males in successive ejaculates. Even better, successive electroejaculations were not detrimental to male guinea pigs’ health because, after completion of the present study, animals returned to the farm and were placed in quarantine, where they displayed healthy behavior and normal physiological constants.

## 5. Conclusions

The present results define macroscopic and microscopic characteristics of guinea pig ejaculates collected by a novel electroejaculation method validated by multiple successive collections from proven fertile males. Semen analysis data, both conventional and flow cytometric, document high efficiency and repeatability of our electroejaculation method. These results can be used as a reference for future comparative studies. Likewise, we recommended fertilization and insemination studies using semen obtained with this technique to facilitate the selection of male guinea pigs for commercial AI service and to improve on existing AI protocols for this economically important species.

## Figures and Tables

**Table 1:** Descriptive statistics of guinea pig’s semen parameters obtained by transrectal electroejaculation.

Parameter	n	Minimum	Maximum	Mean ± SD
Volume (mL)	39	0.10	2.40	0.67 ± 0.5
Motility (%)	39	63.00	99.00	91 ± 6.6
Concentration (×10^6^ spermatozoa/mL)	37	6.00	94.60	36.70 ± 28.4
Total number of spermatozoa (×10^6^ spermatozoa/ejaculate)	37	1.40	60.52	20.09 ± 17.6
Acrosomal defect (%)	39	0.63	21.24	7.80 ± 5.2
Acrosomal absence (%)	39	0.44	25.08	6.51 ± 6.3
Other abnormalities (%)	39	0.25	23.32	3.95 ± 4.5
Total abnormalities (%)	39	3.22	33.95	18.26 ± 8.5
Highly ubiquitinated spermatozoa (%)	37	0.35	31.21	5.57 ± 6.3
Ph	14	7.00	8.0	7.04 ± 0.1

n = Sample size.

**Table 2:** Seminal parameters and ejaculate numbers of guinea pig semen obtained by transrectal electroejaculation.

Parameter	Ejaculate 2		Ejaculate 3		Ejaculate 4	
	n	Mean ± SD	n	Mean ± SD	n	Mean ± SD
Volume (mL)	16	0.5 ^a^ ± 0.5	13	0.6 ^a^ ± 0.4	10	0.9 ^a^ ± 0.8
Motility (%)	16	92 ^a^ ± 6.0	13	88 ^a^ ± 8.8	10	93 ^a^ ± 2.9
Concentration (×10^6^ spermatozoa/mL)	14	34.6 ^a^ ± 27.5	13	40.7 ^a^ ± 31.8	10	34.3 ^a^ ± 27.4
Total number of spermatozoa (×10^6^ spermatozoa/ejaculate)	14	2.6 ^a^ ± 11.3	13	23.3 ^a^ ± 18.5	10	26.2 ^a^ ± 21.2
Total abnormalities (%)	16	16.7 ^a^ ± 7.7	13	18.3 ^a^ ± 9	10	20.5 ^a^ ± 9.3
Ubiquitinated spermatozoa (%)	14	4.8 ^a^ ± 7.8	13	6.1 ^a^ ± 5.9	10	5.9 ^a^ ± 4.7

^a^ Superscripts indicate no significant statistical difference between ejaculates at *p* ≤ 0.05; n = Sample size.

**Table 3:** Comparison of sperm morphological abnormalities in successive ejaculates of guinea pigs obtained by transrectal electroejaculation.

Characteristics	Ejaculate 2		Ejaculate 3		Ejaculate 4	
	n	Mean ± SD	n	Mean ± SD	n	Mean ± SD
Abnormal acrosome (%)	16	6.98 ^a^ ± 4.31	13	7.99 ^a^ ± 4.66	10	8.86 ^a^ ± 7.12
Acrosomal absence (%)	16	5.17 ^a^ ± 5.91	13	7.03 ^a^ ± 6.05	10	7.98 ^a^ ± 7.22
Other abnormalities (%)	16	4.60 ^a^ ± 5.55	13	3.35 ^a^ ± 3.61	10	3.70 ^a^ ± 4.05
Total abnormalities (%)	16	16.75 ^a^ ± 7.79	13	18.37 ^a^ ± 9.04	10	20.53 ^a^ ± 9.31

^a^ Superscripts indicate no significant statistical difference between ejaculates at *p* ≤ 0.05; n = Sample size.

## References

[1] Chauca L. Producción de cuyes (Cavia porcellus) en los países andinos. Food and Agriculture Organization of the United Nations (FAO). http://www.fao.org/3/V6200T/v6200T05.htm.

[2] Garcia C., Moncayo J. (1998). Crioconservación de semen e inseminación artificial en cuyes (Cavia porcellus). Thesis for zootechnist.

[3] Santos V. (2007). Importancia del cuy y su competitividad en el mercado. Arch. Latinoam. Prod. Anim.

[4] Instituto Nacional de Estadística e Informática Perú INEI: IV Censo Nacional Agropecuario 2012. http://censos.inei.gob.pe/Cenagro/redatam/#.

[5] Archetti E.P. (1984). Análisis de la Producción, Formas de Consumo, Comercialización y Simbología del Cuy en Ocho Comunidades de la Sierra Ecuatoriana.

[6] Instituto Nacional de Estadística y Censos. https://www.ecuadorencifras.gob.ec/censo-nacional-agropecuario/.

[7] Encuesta Nacional Agropecuaria ENA: Oferta agropecuaria—cifras 2007. http://www.agronet.gov.co/www/htm3b/public/ENA/ENA_2007.

[8] Ministerio de Asuntos Campesinos y Agropecuarios MACA: Situación de los recursos zoogenéticos en Bolivia 2004. http://www.fao.org/ag/againfo/programmes/en/genetics/documents/Interlaken/countryreports/Bolivia.pdf..

[9] Tecirlioǧlu R.T., Hayes E.S., Trounson A.O. (2002). Semen collection from mice: Electrojaculation. Reprod. Fertil. Dev..

[10] Freund M. (1969). Interrelationships among the characteristics of guinea-pig semen collected by electro-ejaculation. J. Reprod. Fertil..

[11] Scott J., Dziuk P.J. (1959). Evaluation of the electroejaculation technique and the spermatozoa thus obtained from rats, mice and guinea pigs. Anat. Rec..

[12] Vivanco W., Angeles V., Chavez J., Muscari J. (1979). Colección, evaluación y conservación del semen del cuy doméstico (*Cavia porcellus*). An. Cient..

[13] Baska K.M., Manandhar G., Feng D., Agca Y., Tengowski M.W., Sutovsky M., Yi Y.J., Sutovsky P. (2008). Mechanism of extracellular ubiquitination in the mammalian epididymis. J. Cell. Physiol..

[14] Sutovsky P. Ubiquitin and other “negative” biomarkers of sperm quality. Proceedings of the Simposio Internacional de Reproducción Animal y Genomica; Facultad de Zootecnia de la Universidad Nacional Agraria La Molina.

[15] Odhiambo J.F., Sutovsky M., DeJarnette J.M., Marshall C., Sutovsky P. (2011). Adaptation of ubiquitin-PNA based sperm quality assay for semen evaluation by a conventional flow cytometer and a dedicated platform for flow cytometric semen analysis. Theriogenology.

[16] Sutovsky P., Aarabi M., Miranda-Vizuete A., Oko R. (2015). Negative biomarker based male fertility evaluation: Sperm phenotypes associated with molecular-level anomalies. Asian J. Androl..

[17] Tengowski M.W., Feng D., Sutovsky M., Sutovsky P. (2007). Differential Expression of Genes Encoding Constitutive and Inducible 20S Proteasomal Core Subunits in the Testis and Epididymis of Theophylline- or 1,3-Dinitrobenzene-Exposed Rats1. Biol. Reprod..

[18] UK Government (1986). Code of Practice for the Housing and Care of Animals Used in Scientific Procedures.

[19] Swallow J., Anderson D., Harris T., Hawkins P., Kirkwood J., Lomas M., Meacham S., Peters A., Prescott M., Owen S. (2005). Guidance on the transport of laboratory animals.

[20] Green C.J., Knight J., Precious S., Simpkin S. (1981). Ketamine alone and combined with diazepam or xylazine in laboratory animals: A 10 year experience. Lab. Anim..

[21] Smith D.G., Senger P.L., Mccutchan F., Landa C.A. (1979). Selenium and Glutathione Peroxidase Distribution in Bovine Semen and Selenium-75 Retention by the Tissues of the Reproductive Tract in the Bull. Biol. Reprod..

[22] Von Baer L., Hellemann C. (1998). Variables seminales en llama (*Lama glama*). Arch. Med. Vet..

[23] Lòpez A., Soderqüist L., Rodriguez-Martinez H. (1999). Sperm viability in ram semen diluted and stored in three different extenders. Acta Vet. Scand..

[24] Olufunke O.-D., Ajani O.S., Oyeyemi M.O. (2014). Spermatozoa morphology and characteristics of *Spondias mombin* L. (Anacardiaceae) protected male Wistar rats exposed to sodium arsenite. J. Vet. Med. Anim. Heal..

[25] Morales Cauti S., Barrios-Arpi M. (2017). Composición y características de la orina en cuyes (*Cavia porcellus*) con linfadenitis cervical. Rev. Electron. Vet..

[26] Kvist U., Bjorndahl L., Kvist U., Bjorndahl L. (2002). ESHRE Monographs—Manual in Basic Semen Analisys.

[27] Brass K., Palma G. (2001). Inseminacion artificial en la especie equina. Biotecnología de la Reproducción.

[28] Arencibia D., Rosario L. (2009). Consideraciones prácticas acerca de la calidad del semen de conejos aplicado en estudios de toxicología de la fertilidad. Redvet.

[29] Birnabaum D., Hall T. (1961). An electroejaculation technique for rats. Anat. Rec..

[30] Dooley M.P., Pineda M.H. (1986). Effect of method of collection on seminal characteristics of the domestic cat. Am. J. Vet. Res..

[31] Rubio-Guillén J., Quintero-Moreno A., González C., Madrid N., Soto E. (2008). Uso de las pruebas de resistencia osmótica para valorar la funcionalidad espermática en toros. Desarrollo Sostenible de la Ganadería Doble Propósito.

[32] Aitken R.J., Wang Y.-F., Liu J., Best F., Richardson D.W. (1983). The influence of medium composition, osmolarity and albumin content on the acrosome reaction and fertilizing capacity of human spermatozoa: Development of an improved zona-free hamster egg penetration test. Int. J. Androl..

[33] Snyder R.L. (1966). Collection of mouse semen by electroejaculation. Anat. Rec..

[34] Paick S.H., Lee B.K., Yoon S.W., Baek M., Kim H.G., Song E.Y., Lyoo Y.S., Lho Y.S. (2008). Electroejaculation in the male rat. Korean J. Urol..

[35] Hammer C.E., Hafez E.S.E. (1970). The semen. Reproduction and Breeding Techniques for Laboratory Animals.

[36] Busso J.M., Ponzio M.F., Chiaraviglio M., Fiol de Cuneo M., Ruiz R.D. (2005). Electroejaculation in the Chinchilla (*Chinchilla lanigera*): Effects of anesthesia on seminal characteristics. Res. Vet. Sci..

[37] Lawson R.L., Krise G.M., Sorensen A.M. (1967). Electroejaculation and evaluation of semen from the albino rat. J. Appl. Physiol..

[38] Mollineau W.M., Adogwa A.O., Garcia G.W. (2008). A preliminary technique for electro-ejaculation of agouti (*Dasyprocta leporina*). Anim. Reprod. Sci..

[39] Caycedo A. (2000). Contribución al desarrollo tecnológico de la especie. Experiencias investigativas en la producción de cuyes.

[40] White I.G., Hafez E.S.E. (1980). Secretion of the male reproductive tract and seminal plasma. Reproduction in Farm Animals.

[41] Stornelli M. (2007). Evaluación de semen en el gato doméstico: Análisis de rutina y metodologías especiales felino. Rev. Bras. Reprod. Anim. Belo Horiz..

[42] Mortimer S.T. (2000). Practical Aspects about insemination in mammals. J. Androl..

[43] Kerns K., Zigo M., Sutovsky P. (2018). Zinc: A necessary ion for mammalian sperm fertilization competency. Int. J. Mol. Sci..

[44] Kerns K., Zigo M., Drobnis E.Z., Sutovsky M., Sutovsky P. (2018). Zinc ion flux during mammalian sperm capacitation. Nat. Commun..

[45] Glickman M.H., Ciechanover A. (2002). The ubiquitin-proteasome proteolytic pathway: Destruction for the sake of construction. Physiol. Rev..

